# Challenges and solutions to nurse-delivered integrated primary health care in Nelson Mandela Bay

**DOI:** 10.4102/phcfm.v17i1.4873

**Published:** 2025-06-04

**Authors:** Zubrina Baartman, Cornelle Young, Justine C. Baron

**Affiliations:** 1 Department of Nursing and Midwifery, Faculty of Medicine and Health Sciences, Stellenbosch University, Cape Town, South Africa

**Keywords:** primary health care, integration of care, primary health care team, organisational support, collaboration

## Abstract

**Background:**

Delivery of a comprehensive and integrated primary health care service to increase healthcare access, quality, equity and efficiency requires an effective working environment.

**Aim:**

To explore perceptions of primary health care nurses in the Eastern Cape, South Africa, regarding the adequacy of their working environment for integrated primary health care service delivery.

**Setting:**

Selected public primary health care clinics in a subdistrict of the Nelson Mandela Bay metropole, Eastern Cape, South Africa.

**Methods:**

A qualitative descriptive explorative design was used. Semi-structured interviews were conducted with nine nurses working in the selected facilities. Data were thematically analysed.

**Results:**

Availability of members of the multidisciplinary primary health care team, nurse competency, responsiveness and productivity levels compromise integrated primary health care. Service delivery is further negatively impacted by a lack of resources and non-optimal collaboration among members of the primary health care team.

**Conclusion:**

Challenges to rendering an effective integrated primary health care service exist within the primary health care working environment. To significantly increase comprehensive and integrated primary health care service delivery as a quality component of South African healthcare, these challenges need to be addressed.

**Contribution:**

An evidence-based description of aspects of primary health care workspaces that compromise integrated primary health care delivery is provided. This information can be used to improve integrated primary health care services. Integrated services are a prerequisite for the Ideal Clinic Initiative, which is a foundation of the implementation of the National Health Insurance (NHI) scheme for South Africa.

## Introduction

### Background

Primary health care (PHC) not only plays a hugely important role in human health but also contributes to the achievement of Sustainable Development Goal (SDG) 3, which is to ‘ensure healthy lives and promote well-being for all at all ages’.^1^ This goal is a cornerstone for achieving accessible, equitable and quality healthcare.^2,3^ It has been established that the integration of promotive, preventative, curative, rehabilitative and palliative divisions in PHC services, called integrated primary health care (IPHC), yields significantly better health outcomes towards the achievement of SDG3.^3^ Integrated primary health care further fosters equity, healthcare efficiency, access and health continuity, which results in healthier populations, improved quality care and the curbing of health spending across the globe.^4,5^

Many countries, especially lower- and middle-income countries (LMICs) with underdeveloped and fragmented PHC services and a high disease burden, find it challenging to achieve IPHC.^6^ South Africa, compared to similar LMICs such as Brazil, has poorer health outcomes^7^ and in certain provinces such as the Free State, shows little evidence of addressing fragmented healthcare.^8^ The philosophy of PHC was pursued in the South African healthcare system; however, the integration of services remains neglected, leaving health provision fragmented, inefficient, too expensive and inaccessible.^8^ The call is thus to improve IPHC delivery and develop an ideal model for IPHC provision.^4^

Primary health care forms the entry point into the South African healthcare system.^9^ Public PHC services are delivered to 84% of the South African population, with most of the care being nurse driven.^10^ The core standards of the National Department of Health through the Ideal Clinic initiative, provide for the optimal functioning of PHC delivery to communities. The focus is on integrated care as a fundamental element of Ideal Clinic status and a prerequisite for the implementation of the National Health Insurance (NHI).^11^ The Ideal Clinic status favours working environments in PHC that promote health worker satisfaction and subsequent high-quality PHC and IPHC provision, all aiming at improved health outcomes.^11,12^

**FIGURE 1:**
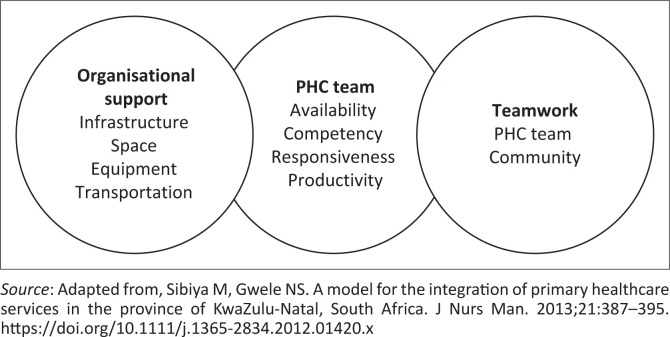
Conceptual model of an enabling working environment for integrated primary health care delivery.

For an enabling environment towards IPHC delivery, certain basic elements illustrated in Figure 1 need to be in place. These include an adequate PHC team, composed of multidisciplinary team members such as doctors, specialist nurses, dieticians, pharmacists, social workers and all allied healthcare providers working collaboratively on the PHC level.^3,5^ Availability, competency, responsiveness and optimal productivity of this team are paramount. The second element includes collaboration of the team, especially on all levels of vertical and horizontal referral, as well as collaboration with the community.^13^ Finally, organisational support is required to provide infrastructure in the form of physical space, transport and equipment.^13^

Reported inadequacy related to all three aforementioned elements in the Oliver Reginald (O.R) Tambo district in the Eastern Cape hindered access to the necessary comprehensive and IPHC.^14^ Such ineffective working environments lead to patient dissatisfaction, compromised quality of care and a loss of trust in the PHC and public health system, clearly impinging on the future goal of an IPHC as a prerequisite for an effective NHI.^15^

To investigate this problem in greater depth, the aim of the research was to explore the perceptions of PHC nurses regarding their challenges and advances towards IPHC delivery during 2022 in a health subdistrict of the Nelson Mandela Bay (NMB) metropolitan area in the Eastern Cape, South Africa.

## Research design and methods

### Study design

A qualitative research method with a descriptive exploratory design was used. The study explored PHC nurses’ perceptions about the capacity of their work environments to support the provision of IPHC services, including PHC team functioning, organisational support and collaboration.

### Setting

The study was conducted at public health clinics in the NMB health subdistrict, in which 1 334 883 inhabitants reside, constituting one-fifth of the total population of the Eastern Cape province.^16^ Three clinics from one of the subdistricts were used. Services are primarily rendered by PHC nurses employed at the clinics. Facilities mainly provide preventative, promotive and curative services, including maternal-and-child-health services, basic antenatal care (BANC), BANC plus, integrated management of childhood illnesses (IMCI), growth and health monitoring, chronic and acute care, tuberculosis (TB) care, human immunodeficiency virus (HIV) counselling and testing and nurse-initiated management of antiretroviral therapy (NIMART). Mental health and youth services are also provided to some degree at some of these clinics. Sessional doctors and dieticians are routinely scheduled for once weekly clinic visits. Ward-based outreach teams (WBOT), consisting of healthcare workers and a nurse as team leader, are assigned to each clinic to provide basic services in the community. Such services entail home visits, directly observed treatment (DOT) of TB medication and community mobilisation during outreaches and clinic campaigns. Primary health care clinic services are rendered from Monday to Friday from 07H30-16H00, excluding weekends and public holidays. The total population served in the month of data collection at the various clinics was 2038 at clinic A, 4219 at clinic B and 3101 at clinic C. All three clinics had Ideal Clinic status at the time of the study.

### Study population and sampling strategy

The study was conducted in government public PHC clinics in the NMB in the Eastern Cape, South Africa. The selection of Subdistrict B and the three clinics was based on the familiarity of the researcher with these clinics as an end user and former employee and a subsequent focus of the researcher to gain more understanding and insight into their working environments and their appropriateness for IPHC delivery.

The population comprised 28 nurses, who came from any category of nurse, and their managers. To be eligible for inclusion in the study, nurses could be permanent or contract Eastern Cape Department of Health (DoH) staff employed at one of the selected PHC clinics. Purposive, non-probability sampling was used to obtain rich information from nurses with relevant experience and knowledge regarding the capacity of their respective workplaces to comply with IPHC.^17^ The sample size was determined by thematic saturation and concurrent data analysis. Including the two pilot interviews, a total of nine participants were interviewed.

### Data collection

Data collection was preceded by the development of a conceptual framework from Sibiya and Gwele’s model^13^ and an interview guide informed by the literature review. The interview guide’s semi-structured questions and prompts were scrutinised for content validity, and questions were modified as necessary. The first author conducted two pilot interviews with PHC nurses in the relevant NMB health subdistrict to test the usefulness of the interview guide and to determine whether the open-ended questions needed modification. The pilot interviews proved to be valuable and were included in the data analysis as part of the study.

Participants’ biographical data, such as age, gender, qualifications, additional PHC training, experience and their position at the workplace, were obtained during the interviews. The opening question explored the functioning of the PHC team for IPHC delivery. Two more broad questions with probes followed on the participants’ experiences of the effectiveness of organisational support and collaboration. All interviews were conducted in English and digitally recorded by the first author and lasted between 18 and 92 min. All but one interview was conducted at the participants’ clinic, and one was conducted at a venue more suitable for the participant. Interviews were transcribed verbatim by the first author, with field notes and summaries that were utilised for confirmation and clarification purposes.

### Data analysis

The analysis was done by the first author and verified for correctness by the second and third authors as supervisors. Professionally transcribed transcripts were compared with audio recordings by the first and second author. Data were analysed using Clarke and Braun’s thematic data analysis method.^18^ Familiarisation with the data as a first step occurred by repeatedly reading the transcripts and relistening to audio recordings and making notes of items of interest.^18^ An Excel spreadsheet was used to store and organise codes. Manual coding was done concurrently with data collection. On completion of the initial coding, codes were grouped together into categories as a third step. For the final step, the themes and subthemes were formulated.

### Trustworthiness

The criteria of credibility, dependability, confirmability and transferability, as suggested by Lincoln and Guba (1985), were used to enhance trustworthiness.^19^ Credibility was promoted via peer debriefing with the research team, who also provided an external check on the research process and perused the audio recordings with the transcripts. Member checking was done by returning each participant’s interview transcript to them with the findings to validate the data interpretation. Data dependability was enhanced by keeping records and an audit trail of the data generation processes, while confirmability was established by checking the findings of the research and verifying that such findings were a true reflection of identified themes and subthemes presented. The first author and data collector had previously worked in PHC clinics for 11 years, which may have influenced personal beliefs, biases, understanding and interpretation of the participants’ responses. To mitigate this, the first author kept a reflexive journal, documenting her reflections, actions and decisions during the research process. Bracketing of preconceptions, together with engagement with the two supervisors, was done to obtain a clear understanding of the research process and ensure integrity in the data analysis and interpretation thereof. Transferability was promoted by providing clear, thick detail of the context and participants as well as the data collection and analysis process to allow other researchers to judge for themselves whether the situations described by the participants and the study methods followed were applicable and relevant to their contexts.

### Ethical considerations

Ethical clearance to conduct this study was obtained from the Stellenbosch University Health Research Ethics Committee 2 (No. S22/05/084) and from the Eastern Cape DoH (EC_202207_013 and RES/BAARTMA/8/2022). The ethical principles of autonomy, justice, beneficence and non-maleficence, determined by the Declaration of Helsinki, were applied in the study.^20^ After permission was obtained from the gatekeepers of nurses in the target population to commence recruitment, a personal invitation to participate in the study was extended to all categories of nurses. The autonomy of participants was ensured through obtaining informed written consent prior to the interviews. To ensure privacy and confidentiality, the identities of the research participants were not disclosed and pseudonyms were used for the interviews, transcriptions and write-up of the study. The transcriber was required to sign a non-disclosure agreement to keep the content of the audio recordings confidential. All the data will be stored in a password-protected database for 5 years.

## Results

The participants’ ages ranged from 27 to 54 years, with nearly half of the sample at 48 years and older. All nine participants were registered nurses, six with registration as a general nurse and midwife and community and psychiatric nursing as part of their basic qualification. The remaining three had completed a 2-year enrolled nurse programme and subsequently the bridging programme to register as general nurses. Participants’ work experience ranged from 9 months to 15 years. Additional PHC short training courses were provided by a district trainer of the Eastern Cape DoH to upskill nurses. None of the clinics had nurses trained to deliver all the necessary services, with shortages of between 6 and 11 functions for such provision (Table 1).

**TABLE 1:** Short courses obtained by nurses in three primary health care clinics (*N* = 3).

Short courses obtained in	Clinic A nurse	Clinic B nurse	Clinic C nurse
PHC certification	2	2	1
BANC (basic antenatal care)	2	2	1
BANC plus (postnatal care)	0	1	0
Breastfeeding	0	0	1
EPI (expanded programme of immunisation)	2	3	1
IMCI (integrated management of childhood illnesses)	2	2	2
Growth and health monitoring	0	1	0
TB (tuberculosis) management	1	2	3
NIMART (nurse-initiated management of antiretroviral therapy)	1	0	0
HIV (human immunodeficiency virus) counselling and testing	1	0	3
Adherence (to HIV therapy) counselling	1	1	0
Prep (pre-exposure prophylaxis treatment)	0	1	0
STI (sexually transmitted infections) treatment	1	0	0
Palliative care	1	0	0
Contraceptive care	1	0	0
Chronic care	1	0	0
Cervical cancer screening (Pap smear course)	0	1	0
COVID-19 vaccination	0	1	0

*Source:* Baartman Z. Perceptions of primary healthcare nurses about the effectiveness of their working environment for integrated primary healthcare services delivery in Nelson Mandela Bay Health District [MA thesis]. Cape Town: Stellenbosch University; 2024

PHC, primary health care; COVID-19, coronavirus disease 2019.

**FIGURE 2:**
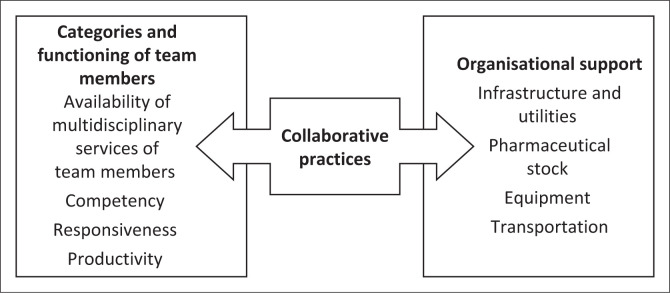
Themes and subthemes.

Three themes were generated from the data, namely: (1) categories and functioning of team members, (2) organisational support, and (3) collaborative practices for a working environment to deliver IPHC (Figure 2) and are discussed as follows.

### Theme 1: Categories and functioning of team members

From the data, the availability of multidisciplinary team members and their competency, as well as the responsiveness and productivity of nurses as drivers for enabling IPHC provision, emerged as a main theme.

#### Subtheme 1.1: Availability of multidisciplinary services of team members

When staff shortages occurred, nurses were obligated to fulfil additional roles in the absence of other multidisciplinary team members or make alternative arrangements with patients. Qualified pharmacists were not employed in the three facilities, and pharmacy assistants were responsible for medication dispensing. One facility used a general worker (whose actual job description was housekeeping, cleaning and general duties) to pack medication for dispensing, and patients seen on a specific day were asked to return the next day for their prescribed treatment, which defeats the purpose of IPHC. The following quotations illustrate these issues:

‘We had a pharmacist before. And then that pharmacist was taken out … [*to a community healthcare centre*] …’ (Participant 4, 27 years, female)

‘He [*pharmacist assistant*] gets help from a GA [*general assistant*] that works with him, that helps him pack medication.’ (Participant 9, 29 years, female)

‘… the chronic patients, we have to say to them, you drop your card [*leave your patient file/appointment card with the clinic*] and then you get your treatment [*fetch it*] the following day.’ (Participant 4, 27 years, female)

Sessional doctors and dieticians visited the clinic on a weekly basis. However, decreased doctor’s visits were reported, causing an overload of patient bookings on the doctor’s visiting day, which meant longer waiting times for patients or extended appointment dates. One of the participants reported:

‘Every Monday, we have a doctor come in; it used to be [*also*] on Friday. But we only have the doctor coming once a week. So, our doctor is overbooked because we have so many people that needs to see the doctor.’ (Participant 1, 35 years, female)

Mental health services were neglected as patients needed referral to psychologists or psychiatrists for initiation of treatment, as explained by one of the participant:

‘For a psychologist [*consult*] we have to go via the doctor. The physicians [*also*] see the mental health patients [*initially*] to order drugs.’ (Participant 3, 48 years, female)

Dietetic services were rendered on a visiting basis, usually once a week. However, when the dietician was engaged at another clinic, a PHC nurse, in addition to her own workload, would have to assume this role as well, as illustrated by Participant 3:

‘She [*the dietician*] had the breast week month, so she did not pitch … She will tell us: “Listen I have got a patient that is coming please assist to give the patient this and that [*supplements*]”, and then we usually act on her behalf.’ (Participant 3, 48 years, female)

Although functioning referral systems between clinics and higher levels of care contribute to effective multidisciplinary teams, the data showed that such services were not integrated at clinic level. For instance, a person living with HIV (PLHIV) who needed a mental health or social work referral would have to travel to another facility (e.g., the community health centre) for the next level of care after receiving their ART:

‘The patient is on ART [*supplements*] and is also on mental health [*supplements*]. So now the patient goes to two different facilities …we can’t integrate those services.’ (Participant 4, 27 years, female)

Nurse category availability was also below the prescribed norms and standards, as noted by the following participants:

‘… at the moment we are very short staffed. One [*PHC nurse*] is resigning in November, she is finishing off. Then two at Psyche [*mental health services*] also finish[*ing*] off in this year, and then two had to go from the floor [*from providing PHC*] to Psych [*provide mental health services to replace the original two who left*]. So basically, there’s only three sisters [*instead of the five before*].’ (Participant 8, 38 years, female)

‘We don’t have a staff nurse [*two years basic nurse training*], and then there is the assistant nurse [*one-year basic nurse training*] in the observation room [*doing the work of both these categories*].’ (Participant 6, 54 years, female)

A lack of clerks and cleaning staff compelled registered nurses to manage the tasks of these job categories, with less availability for IPHC, as illustrated in the following quotes:

‘… we do not have clerks, we start at reception in the morning, and we take out files [*ourselves*].’ (Participant 3, 48 years, female)

‘… they said they do not know when they are going to get a cleaner for us. Coming in the rooms, we must clean it ourselves. The garbage bags, we must empty it ourselves. So, it delays the service that we are supposed to be doing.’ (Participant 3, 48 years, female)

#### Subtheme 1.2: Competency

Staff competency is influenced by the availability of adequately qualified candidates for recruitment, as well as the knowledge and skills of the currently employed staff. New appointments were reported to be very limited, possibly because of austerity measures, and clinics had to make do with the staff who often lacked the required IPHC skills. Lack of retention of adequately trained nurses had led to these tasks and functions being performed by nurses who had been trained to the required level and were ill-equipped. This had a negative effect on integrated health care:

‘If I [*community service fully qualified registered nurse*] go, they will be short-staffed. I am on contract [*until end of year*], I’m not permanent. They are no longer absorbing now.’ (Participant 9, 29 years, female)

‘We don’t have a staff nurse and then there is the assistant nurse [*ENA*] in the observation room [*replacing the enrolled nurse or staff nurse*].’ (Participant 6, 54 years, female)

Not only were pharmacy assistants and cleaners offering services for which they were not trained, nurses were also working outside their scope of practice. Some nurses were unable to deliver the required services as they had not had sufficient training. With a constant shortage of staff, upskilling was problematic as services could not be interrupted or halted to allow for training. Scheduled training sessions were often cancelled, depriving staff of the opportunity to perform better in the delivery of IPHC:

‘Currently, I am in the primary health care setting, [*without training*] and I do consultation of patients [*acute and chronic patients*] …’ (Participant 7, 54 years, female)

‘Since I’m dealing with the chronic patients, so I would like to also have that kind of information [*obtained through chronic care training*].’ (Participant 4, 27 years, female)

‘But then there are those days when the trainings are cancelled for one or the other reason. Or we are so little on duty, that we cannot be sent to the trainings. Most of the training is in PE [*now Gqeberha, not in the immediate area*] and then there is transportation challenges that we face [*to get to the training*].’ (Participant 6, 54 years, female)

#### Subtheme 1.3: Responsiveness

Responsiveness is the ability to deliver a ‘one-stop shop’ experience to the satisfaction of the client and where the client does not have to return for services unnecessarily. At the clinic level, nurses were unable to adequately respond to certain needs and to deliver IPHC because of a lack of training, as illustrated in the following quotes:

‘… you have the sisters that can’t work antenatal and … EPI and IMCI because they are not feeling comfortable, because it’s been a very long time since they worked there.’ (Participant 1, 35 years, female)

‘We train each other. But it does not really happen on regular basis. Because there is no time.’ (Participants 3, 48 years, female)

High patient loads, because of providing services to patients from other catchment areas, meant that nurses were often unable to take necessary tea and lunch breaks, and worked after official working hours to assist patients who needed higher levels of care. This indicates good responsiveness but could result in forms of negligence and the frustration of not receiving recognition or any form of compensation. To illustrate:

‘We’re not only serving our catchment area. We get people from all other areas …’ (Participant 8, 38 years, female)

‘I especially need someone to assist me in the baby room. Because at the moment tea is out for me.’ (Participant 8, 38 years, female)

‘Everything pile[*s up*] … and the pressure is just put on and there is no compensation [*time, money*] for anything.’ (Participant 7, 54 years, female)

#### Subtheme 1.4: Productivity

To manage the workload, staff had to take shortcuts, neglect or skip some work, miss training sessions and remain in the specific clinic service instead of rotating (eventually unlearning some skills). This, together with absenteeism because of exhaustion, influences productivity, resulting in an extended waiting times for the patients, as illustrated by the following quotations:

‘… it’s just sometimes when the clinic is very busy, then you don’t always get the time to do everything properly.’ (Participant 1, 35 years, female)

‘If I can talk about myself for the last year [*or*] half of the year, I didn’t feel like coming on duty, I’m tired.’ (Participant 6, 54 years, female)

‘Most of the time patients feel that they are waiting too long in the queues to be served.’ (Participant 7, 54 years, female)

### Theme 2: Organisational support

Organisational support includes providing and maintaining the necessary infrastructure, space, utilities, pharmaceutical supplies, transport and equipment.

#### Subtheme 2.1: Infrastructure and utilities

The dilapidated state of clinic buildings was a source of frustration, as facilities were old, small and inadequate for IPHC delivery. Examples of this included lack of building maintenance, insufficient ventilation in the TB room, not enough handwashing facilities and lack of privacy as no screens were available. Examples of this were offered by participants as follows:

‘Then the waiting area here by us is too small, because antenatal, children, acute, doctors’ patients [*are all waiting*] all in one room, so … In the morning, people need to sit outside, as there’s no space into the waiting area. And sometimes it rains and it’s cold so then you [*the patient*] can decide yourself [*whether to wait in those conditions*].’ (Participant 5, 41 years, female)

‘There is a lot of cracks in the building and leakages, geysers were not working, because we do not have hot water. There is a need for painting, walls to be cleaned or whatever, windows, but no one comes back [*to fix-up the situation*].’ (Participant 3, 48 years, female)

To function properly, clinics need clean running water and electricity. Alarmingly, some facilities sporadically lacked clean running water and suffered from continuous sewerage problems. Consequently, for weeks at a time, the PHC staff of these facilities had to make alternative ablution arrangements during working hours, with no such options for patients:

‘Two weeks ago, they stole off the pipes and then there was no water for a week, and … obviously there were no toilet services, you couldn’t really wash your hands. Next door is a home for the challenged … they do have toilets there. Then we can go one at a time if the need is like that.’ (Participant 6, 54 years, female)

Some consulting rooms (those without windows) would become too dark to work in during loadshedding. Patients were requested to return for services after loadshedding, which resulted in patient discontent and possible non-adherence:

‘This morning, I could not do the pap smear because there was no light, and it becomes very dark. So, I had to re-book this lady to come tomorrow or maybe a little later, because the load-shedding was going to last two hours. She was not happy at all. So, basically, it is a chain of terrible service because of no electricity.’ (Participant 3, 48 years, female)

#### Subtheme 2.2: Pharmaceutical stock

Stock-outs of pharmaceutical supplies occurred regularly at clinics. Patients would then be requested to collect their medication from other health facilities, or they had to make a return visit for the collection of medication:

‘We face challenges of certain resources that is not to our immediate disposal. And therefore, it does have a negative impact on how we treat our patient … Because you will find that in the case where you have to prescribe medication for the patient, that specific medication is not available.’ (Participant 7, 54 years, female)

#### Subtheme 2.3: Equipment

A shortage of the necessary diagnostic equipment was also experienced, with some nurses having to share haemoglobinometers (HB meter), baumanometers, haemoglucotest machines, otoscopes, thermometers, scales and dopplers. In the absence of an HB meter, expensive blood tests had to be performed, or staff would have to rely on physical assessments only by using their senses (looking, listening, touching), without confirming evidence for diagnosis. To illustrate:

‘… sometimes we don’t have an HB meter. So now we have to take bloods. And maybe you want that reading now [*to make a diagnosis, but it is only available the next day*]. But when the HB meter is not working, now, we have to give [*vitamin*] supplements without knowing how the HB is.’ (Participant 4, 27 years, female)

‘And then also not all the rooms have ENT sets [*Ear-Nose and Throat – thus otoscopes*] so you have to move from one room to another. We only have one BP machine [*baumanometer*]. So, you have to wait for another patient to finish [*having blood pressure taken*] before you can do [*your patient’s*] blood pressure.’ (Participant 1, 35 years, female)

#### Subtheme 2.4: Transportation

The only official transport available to patients who were referred to the next level of care was the emergency medical service. Patients sometimes had to wait several hours for such transport, often until after the clinic was formally closed for the day. Those patients who did not want to wait and had the means to go to referral centres by private or public transport (minibus taxis) had to leave without the necessary healthcare staff support. In one urgent case, a PHC nurse had accompanied a very sick baby to the hospital in the patient’s private vehicle and had to return to work with a taxi at her own cost. Such a lack of efficient transport caused a disruption in the referral of patients to higher levels of care:

‘Ambulance services, you would phone that number ten times, they are not responding. I actually went with the baby with the [*patient’s private*] transport the one time [*to secondary service to help, due to the urgency of the situation*].’ (Participant 8, 38 years, female)

### Theme 3: Collaborative practices for a working environment to deliver IPHC

The availability of a full complement of team members and adequate infrastructure makes collaboration possible. It became evident, in the first two themes, that this availability was lacking, and collaboration between the nurses, other multidisciplinary team members and facility management was less than ideal. In one case, team members were reported to have screamed at one another in front of patients. Such behaviour has the potential to decrease collaboration even more, and it is unsettling to the community members who observe the confrontation:

‘… people have their own attitudes and their own way of sorting out things and how they handle things … Sometimes they [*staff members*] are screaming at each other.’ (Participant 8, 38 years, female)

Yet, participants showed their understanding and appreciation in those areas where they recognised successful collaboration, as explained by some of the participants:

‘We’re working together as a team, really, we do. Where there is an emergency, then all of us go. And then we help each other dealing with it.’ (Participant 2, 51 years, female)

‘Shame, they work very hard, they are very productive.’ (Participant 4, 27 years, female)

Often, PHC nurses perceived management and patients to be unappreciative and felt frustrated, ignored and undervalued. This was evident from the following quotations:

‘So, at ground level sometimes you feel frustrated that [*y*]our voices are not heard, and we are ignored … there is no sense of appreciation from the management … and at times from the patients.’ (Participant 3, 48 years, female)

‘Our in-charge [*facility manager*] is very easy to speak to. But as soon as you go higher … although they said if there’s any problems, you can just phone but really, we are not getting the support from the management.’ (Participant 1, 35 years, female)

The effectiveness of clinic-community partnerships was evident in facility operations. Functional clinic committees provided guidance to clinics and facilitated communication with communities coping with substandard services, which helped to identify needs and options for improvement of IPHC services:

‘Clinic committee members, they have just come in [*use*] now, but recently, they were quite helpful … they have been attending to the complaints, compliments, and all that, and so far, they are new, but we can feel their presence …’ (Participant 3, 48 years, female)

## Discussion

The study findings revealed numerous shortcomings in the working environment for IPHC delivery in the NMB health subdistrict. Such shortcomings included the lack of availability of all members of the multidisciplinary team at PHC level, especially shortages of all categories of nurses. Mabunda et al.^22^ confirm that health worker shortages have direct bearing on nurses, as PHC service delivery is nurse led. Tshililo et al.^23^ similarly report on the unavailability of staff as a hindrance to PHC service delivery. The shortage of nurses is exacerbated by a shortage of pharmacists, doctors and social workers on the team. As a result, nurses must often take on responsibilities beyond their primary role. Without the necessary qualifications and competencies, they are working outside their scope of practice. This places patients and providers at risk, despite nurses being willing and often confident to extend their roles. This also exposes the nurse to litigation if an adverse event occurs. Continuous and life-long in-service training is a possible solution for facilitating and enhancing integrated care at PHC level.^24,25,26^

A Brazilian study has shown that the widespread burnout among healthcare professionals and PHC teams can lead to medical malpractice and increased health system costs.^27^ Nurses in the current study acknowledged their challenges and the effects of inadequate organisational and infrastructural support (utilities, pharmaceutical stock, equipment and transport). Other studies have also highlighted the slow progression of the South African government to regenerate existing healthcare infrastructure.^15,28^ This insufficiency of utilities in health facilities fails to meet the World Health Organization (WHO) action plan, which stipulates that the provision of water, sewerage and hygiene (WASH) services at all health facilities is a crucial aspect of healthcare delivery.^29^

The reported drug stock-out situation incurred recurring clinic visits for patients and had a negative impact on service delivery. Similarly, a Vietnamese study cited a lack of medication in both urban and rural areas but mentioned that those who could afford healthcare did self-referrals to secondary levels of care to avoid the medication shortfall.^30^ In South Africa, secondary levels of care are overloaded because of dissatisfied PHC clients who cannot receive adequate treatment at primary level facilities. Extended waiting times have led some patients in the Harry Gwala district, KwaZulu-Natal, to leave PHC facilities without receiving care. This increases the risk of non-adherence and places additional strain on the next level of care.^31^

The transportation of sick patients to referral sites took place haphazardly, jeopardising the continuity of care and IPHC delivery. The findings align with studies conducted in KwaZulu-Natal and the Tswane district in Gauteng that reported that unpredictable ambulance services prolong the transfer of patients to referral centres.^31,32^

Healthcare users from Spain, Australia and Canada have reported challenges regarding collaboration among multidisciplinary team members.^33,34,35^ Interventions from the Australian and Canadian governments were sought to improve collaborative practices between PHC team members to promote patient satisfaction, improve health outcomes and strengthen the PHC system.^34,35^

There are ethical concerns for facility management in settings where nurses provide care and treatment outside their scope of practice. Poor vertical (nurses with management) and horizontal (collegial) relationships and collaboration result from continued experience of a lack of management support, as shown in the present study. This corresponds with a study from KwaZulu-Natal that identified inadequate communication skills of managers as the reason for poor engagement with and between staff members.^36^ Similarly, in Ekurhuleni, South Africa, the lack at all levels of management support was the reason provided for substandard health provision.^37^

Clinic-community relations and collaboration were advanced to support PHC clinics. A customary practice in Lesotho is that PHC nurses liaise with community leaders, community health workers and non-profit organisations to enhance community participation.^38^ In contrast to these findings, in Johannesburg PHC team members perceived that their workloads increased as a result of community mobilisation. This resulted in disagreements between members of outreach teams and PHC staff in the clinics.^39^ Such disagreements have also spilled over to impact on the collaboration between clinic staff and the community and/or patients.

## Recommendations

Strategies to recruit and retain healthcare professionals in the PHC system are crucial, such as the absorption of newly qualified professional nurses into PHC services and dedicated non-negotiable time set aside for upskilling of all nurses expected to deliver IPHC. Training and upskilling programmes should be offered at the facilities, rather than removing nurses from the workplace. Opportunities for further study should be provided to nurses to obtain the specialist, postgraduate primary care qualification via skills development and training programmes. These programmes should be regularly reviewed to ensure that training needs are addressed and that all PHC team members receive an equal opportunity for professional development.

Deliberation should take place with the South African Pharmacy Council to broaden the scope of practice of pharmacy assistants in the absence of pharmacists. In addition, unauthorised and unqualified personnel (such as general assistants or cleaners) should not be permitted to perform any pharmaceutical duties.

Staff support, debriefing sessions and strengthening wellness at work programmes are necessary to alleviate burnout and stress in the workplace. Regular surveys about the quality of work–life could also improve the situation if the results are acted upon.

It is proposed that patients receive timeous care at facilities and within acceptable, prescribed waiting times, according to the Ideal Clinic manual.^11,40^ In addition, the inadequate infrastructure at PHC facilities requires that national authorities revise action plans for infrastructure development and maintenance in preparation of NHI implementation. Priority should be given to ensuring a safe workplace environment through increased security measures.

The transportation of patients by ambulance personnel to facilities with higher levels of care also need attention. Deliberation should take place on how to overcome challenges faced by both ambulance and PHC services to find a workable solution for rendering prompt referral services when needed. The use of other, safer forms of patient transport should be explored to relieve the financial burden of patients who do not require same day referral.

## Limitations of the study

The data for this study were generated from one of three subdistricts and only 3 clinics out of 15 from the relevant health subdistrict. The researcher tried to avoid bias in the study; however, some reticence was noticed from few of the participants to elaborate on problems encountered at their workplace. This can be because of the sensitive nature of these issues. Probes were necessary to generate a greater depth of information, all which needed to be verified and checked for accurate interpretation.

## Conclusion

This study explored the capacity of the working environment for IPHC delivery from the perspectives of nurses working in PHC clinics. These nurses identified that, at the time of the study, their working environments in the NMB, health subdistrict B in the Eastern Cape, were not conducive to rendering an effective IPHC service. The importance of a full multidisciplinary team, available, competent and responsive nurses, adequate organisational support and collaboration among the team members, vertically and laterally, for effective IPHC functioning has been demonstrated. The study further revealed the present weaknesses in the PHC services. Comprehensive integrated and quality PHC service delivery, as a quality component of South African healthcare, is imperative for NHI implementation.
